# Bio-Activation of HA/β-TCP Porous Scaffolds by High-Pressure CO_2_ Surface Remodeling: A Novel “Coating-from” Approach

**DOI:** 10.3390/ma15207306

**Published:** 2022-10-19

**Authors:** Clémentine Aubry, Christophe Drouet, Thierry Azaïs, Hyoung-Jun Kim, Jae-Min Oh, Ipek Karacan, Joshua Chou, Besim Ben-Nissan, Séverine Camy, Sophie Cazalbou

**Affiliations:** 1 Centre Inter-Universitaire de Recherche et d’Ingénierie des Matériaux, CNRS/UT3/INP, Université de Toulouse, 31030 Toulouse, France; 2 Laboratoire de Génie Chimique, CNRS/UT3/INP, Université de Toulouse, 31030 Toulouse, France; 3 ARN: Régulation Naturelle et Artificielle, INSERM U1212, CNRS, Université de Bordeaux, 33076 Bordeaux, France; 4 Laboratoire de Chimie de la Matière Condensée de Paris-UMR 7574, CNRS, Sorbonne Université, 75005 Paris, France; 5 Department Energy and Materials Engineering, Dongguk University, Seoul 04620, Korea; 6 Research Institute, National Cancer Center, Goyang 10408, Korea; 7 University of Technology Sydney, Ultimo 2007, Australia

**Keywords:** BCP, bioactivation, surface remodeling, supercritical CO_2_, characterization, antibacterial, “coating from” approach

## Abstract

Biphasic macroporous Hydroxyapatite/β-Tricalcium Phosphate (HA/β-TCP) scaffolds (BCPs) are widely used for bone repair. However, the high-temperature HA and β-TCP phases exhibit limited bioactivity (low solubility of HA, restricted surface area, low ion release). Strategies were developed to coat such BCPs with biomimetic apatite to enhance bioactivity. However, this can be associated with poor adhesion, and metastable solutions may prove difficult to handle at the industrial scale. Alternative strategies are thus desirable to generate a highly bioactive surface on commercial BCPs. In this work, we developed an innovative “coating from” approach for BCP surface remodeling via hydrothermal treatment under supercritical CO_2_, used as a reversible pH modifier and with industrial scalability. Based on a set of complementary tools including FEG-SEM, solid state NMR and ion exchange tests, we demonstrate the remodeling of macroporous BCP surface with the occurrence of dissolution–reprecipitation phenomena involving biomimetic CaP phases. The newly precipitated compounds are identified as bone-like nanocrystalline apatite and octacalcium phosphate (OCP), both known for their high bioactivity character, favoring bone healing. We also explored the effects of key process parameters, and showed the possibility to dope the remodeled BCPs with antibacterial Cu^2+^ ions to convey additional functionality to the scaffolds, which was confirmed by in vitro tests. This new process could enhance the bioactivity of commercial BCP scaffolds via a simple and biocompatible approach.

## 1. Introduction

Orthopedics, cranial and maxillofacial reconstructions represent a major sector of surgical procedures at present. Furthermore, the aging of the population in recent decades has generated a significant increase in patients needing skeletal repair [1,2]. This led to an increase in age-related pathologies and accentuated skeletal fragility, as well as problems of dependence when there is a physical functional limitation (e.g., due to skeletal diseases or traumas) with an inability to perform daily life activities. Engineered biomaterials are developed as bone substitutes, which are potentially multifunctional, to improve the quality of patients’ life and to allow for them to recover normal physical activity as quickly as possible.

For bone repair, although autologous bone graft has long been considered the gold standard, it has limitations in terms of amount of available graft tissue and it increases the morbidity and risks of surgical site infection and pain. Instead, the development of synthetic bone substitutes is now clearly favored, in combination with several advantages such as unlimited resources, the absence of transferred pathogens and the possibility to tailor their shape, size and composition to address specific clinical needs. Among bone biomaterials, calcium phosphates (CaP) and bioactive glasses are by far the two main groups of compounds used at industrial and clinical scales [3,4,5]. CaPs are directly inspired by the composition of natural bone, which involves ca. 70 wt.% of a CaP apatitic phase [6], and, as such, they exhibit intrinsic biocompatibility, making them very adequate for bone regeneration purposes. Among CaPs, the development of high-temperature and sintering processing approaches [7] has favored the development of well-crystallized high-temperature phases, such as stoichiometric hydroxyapatite (HA, Ca_10_(PO_4_)_6_(OH)_2_) and β-tricalcium phosphate (β-TCP, Ca_3_(PO_4_)_2_) [8,9,10]. One limitation of HA is its very low solubility (pK_sp_~117 for the Ca_10_ stoichiometry) [11,12], leading to poor ion release kinetics (and related bioactivity) and a non-resorbable character. Combinations of HA with β-TCP could seen as a way to artificially increase the resorption rate of BCPs. Indeed, although β-TCP has a solubility product (pK_sp_~29) [13] greater than that of body fluids such as blood (pK_sp_~25–26) [14], this CaP phase is rather readily resorbable upon osteoclast cell activity [15]. BCPs are commonly produced commercially and used clinically in orthopedics and dentistry—e.g., in the form of porous or dense scaffolds—for filling bone defects (e.g., after bone tumors or traumas), and to provide support for bone healing in prosthesis revision and joint arthrodesis [16,17]. Such BCPs have several appealing assets such as ease of fabrication (despite the need to generate high temperatures) in different shapes and with tailorable porosity, as well as relevant mechanical properties, especially for bone sites under moderate pressure solicitations. However, these two high-temperature phases, HA and β-TCP, although osteoconductive, present limited bioactivity related to their low specific surface areas and poor ability to release ions. Contrary to natural bone mineral (or its biomimetic synthetic analogs) [6,18,19], these phases do not allow for fast ion exchanges with body fluids or extensive molecular adsorption possibilities to potentially accelerate bone regeneration.

Strategies are increasingly being explored to increase biomaterials’ bioactivity, which may occur via bioactive calcium phosphate phases. Such strategies gather “bio-activation” approaches, aiming to favor natural healing pathways and increase biomedical potentialities by conveying additional properties (e.g., antimicrobial) [20,21]. Concerning BCPs, the “bio-activation” strategies have been based on the deposition of more bioactive CaP phases, leading to a bio-inspired coating capable of improving the biological behavior of the scaffold [22]. Wet processes allowed, for example, to deposit biomimetic apatites or their reactive hydrolysable precursors such as brushite (dicalcium phosphate dihydrate, DCPD, CaHPO_4_.2H_2_O) or octacalcium phosphate (OCP, Ca_8_(PO_4_)_4_(HPO_4_)_2_.5H_2_O). A significant amount of research has been conducted in this direction, inspired by work such as that from Kokubo’s group [23]. The substrate was covered by a highly reactive apatite layer formed from a metastable solution. Agents such as CO_2_ can then be used to modify the pH of the solution and modify the composition of the precipitated phases [24]. Such a coating process has the advantage of allowing for the treatment of surfaces of complex shapes but suffers from difficulties related to their use at an industrial scale, with uncontrolled phases’ precipitation being the main issue. Other works have proposed to coat porous BCP ceramics with a reactive “nanoporous” biomimetic apatite coating using an impregnation-drying process from a calcium phosphate nanogel [25,26]. The presence of a biomimetic apatite coating on the BCP porosity allowed for significant increase in the molecular adsorption capacity of the ceramic (illustrated with the BMP-2 growth factor) while also providing a net increase in new bone formation and unveiling osteoinductive properties—which was not observed in stoichiometric HA nor β-TCP. Biomimetic apatites showed great bone regeneration capabilities in vivo [26,27,28], ich was wh not reached with high-temperature CaP phases. This coating approach has some limitations, especially regarding the adhesion of the coating on the substrate.

Consequently, at this stage, several of the studies cited above have reported that exposing biomimetic apatite (or its metastable/hydrolysable precursors such as OCP) on BCP surface led to a clear increase in in vivo bone neoformation and enhanced significantly the osteointegration of the implants by conferring an osteoinductive character. The technological challenge that remains lies in the strategy to successfully expose such bioactive CaPs compounds at the surface of BCPs while preserving the overall integrity of the scaffolds, not blocking their porous network, without a lack of adherence to the substrate, and with an easily industrializable process.

Furthermore, bacterial infections are a major concern in orthopedic and dental surgery [29,30]. While the use of antibiotics might generate bacterial resistance mechanisms [31], some ionic species, such as copper ions (Cu^2+^), are well known for their antibacterial intrinsic properties [32]. Copper ions thus proved to be very relevant in the biomedical field and bone engineering thanks to their antibacterial and pro-angiogenic properties, as well as for their ability to potentially stimulate osteoblast cell activity [33,34].

In the present work, we have conceptualized a novel strategy to address these questions, based on an original coating-from instead of a coating-to approach. In contrast to depositing an external CaP coating, this concept lies in the direct chemical modification of the surface of BCPs upon treatment with high-pressure CO_2_ (using a supercritical CO_2_ reactor). CO_2_ is particularly relevant for its three functions of (1) pH modifier, (2) carbonate ions’ provider and (3) sterilizing agent. Moreover, various industrial processes already use high-pressure CO_2_, for example, as a “green” and biocompatible solvent [35], including for biomedical applications [35,36,37], evidencing clear scale-up possibilities.

The innovative approach of this study resides in the use of high-pressure (supercritical) CO_2_ to allow for water-immersed BCP surface remodeling. In simple terms, this may be seen as a “*coating-from*” rather than a “*coating-to*” approach, as no addition of external solid substance is needed, conversely to traditional coating strategies [22,23,25]. Such a strategy could thus be applied to existing, commercial BCPs, in order to significantly enhance their bioactivity and renew their clinical relevance.

We will here describe this proof-of-concept and validate it on actual commercial BCPs. We will explore the properties of the newly formed phases using complementary techniques such as X-ray diffraction (XRD), Fourier transform infrared spectroscopy (FTIR), scanning electron microscopy (FEG-SEM), solid-state Nuclear Magnetic Resonance (NMR), specific surface area measurements (BET) and mercury intrusion porosimetry, as well as indirect tests as ion exchange experiments. We will then evidence the possibility of doping such remodeled BCPs surfaces with bioactive Cu^2+^ ions and demonstrate their antibacterial features toward two major bacterial strains, as well as the absence of acute cytotoxicity to osteoblast cells.

## 2. Materials and Methods

### 2.1. Materials

The present study was carried out on cubic-shaped, commercial macroporous BCP scaffolds (Ceraform^®^, Teknimed, L’Union, France) of 3 mm height, consisting of HA/β-TCP (65/35 wt.%) and with a pore diameter in the range 100–400 µm (65% porosity). HA powder, used as a reference in this work, was provided by the Teknimed Company (100% pure HA, calcined at 750 °C, n° 8011011).

### 2.2. Supercritical CO_2_ Process

A supercritical cell (E3100) from Quorum Technologies, Lewes, UK, was used to perform high-pressure CO_2_/water treatments. The cell temperature was regulated thanks to a thermostated bath (Isotemp Fisher Scientific-Waltham, MA, USA).

In the “reference” protocol used, unless otherwise specified in the text, the following experimental conditions were applied. Before the treatment, the ceramics were immersed into deionized water with a liquid/solid (L/S) mass ratio of 2:1 and the mixture was inserted into the supercritical CO_2_ cell. The chamber was filled at 5 °C with liquid CO_2_ at the bottle pressure, before being heated to 37 °C. Upon this temperature rise, the pressure increased to reach 80 bar. The system was maintained at 37 °C/80 bar for 4 h to allow for BCP surface modification/stabilization, then slowly depressurized for 30 min. After this treatment, the BCP/water mixture was extracted from the device and left to equilibrate for 1 h of “resting time” at room temperature, while still in contact with the medium. The ceramics were then retrieved, washed with deionized (DI) water and oven-dried 24 h at 50 °C.

### 2.3. Physicochemical Characterizations

Different methods were used to characterize the surface modifications that the CO_2_-treated BCP scaffolds underwent.

X-ray diffraction (XRD) analyses were carried out using a Bruker AXS GmbH, Karlsruhe, Germany) with Cu Kα radiation (λ = 1.5406 Å) from 2θ = 0 to 80° (2 h acquisition). XRD data were processed with the Match! 1.11 software(version 3.7.1.132, Crystal Impact, Bonn, Germany). The Eva^®^ software (version 5.1, Brucker, Billerica, MA, USA) was used to evaluate, via Relative Intensity Ratio (RIR) refinement, the amount (wt.%) of crystalline phases before and after CO_2_ treatment.

Fourier-transform infrared (FTIR) spectra were recorded using a PerkinElmer 1700 spectrometer (Thermo Fisher Scientific, Waltham, MA, USA) in the wavenumber range 4000–400 cm^−1^ at a resolution of 2 cm^−1^. The analysis was made in transmission mode with the KBr pellet method. Data were analyzed using the OMNIC 9.6.251 software.

Morphological characterization was carried out using scanning electron microscopy (SEM) using a FEG-SEM JSM 7100F TTLS (Field Emission Gun, JEOL, Tokyo, Japan). To limit electron evacuation artifacts, the samples were preliminarily coated with a 9 nm layer of gold thanks to a SI50B sputter-coater (Edwards, Crawley, UK) for 90 s.

The specific surface area was evaluated via the Brunauer, Emmett and Teller (BET) method using an ASAP 2010 (Micromeritics, Norcross, GA, USA) apparatus. Krypton gas was used due to the very low specific surface area of the initial BCP ceramics (<1 m^2^/g) with a measurement error of 0.03 m^2^/g.

Mercury intrusion porosimetry (AutoPore III, Micromeritics Instruments Inc., Norcross, GA, USA) was used to access the inner characteristics of the porous network between 360 and 0.003 nm. The pore size distribution was calculated as the intrusion differential volume of mercury plotted against the pore size.

To further explore the present CaP phases, solid-state NMR spectroscopy analyses were performed on treated and untreated BCP ceramics. Solid-state NMR spectra were conducted on a Bruker 300 MHz Avance-III spectrometer (7.0 T, Billerica, MA, USA) operating at ν(^1^H) = 300.29 MHz and ν(^31^P) = 121.56 MHz. Powdered samples were packed into 4.0 mm (o.d.) zirconia rotors and spun at 5 kHz in a 4BL CP/MAS 1H/BB probe. 31P single-pulse MAS solid-state NMR experiments were recorded using a recycle delay (RD) of 200 s and a 30° flip angle. ^1^H → ^31^P cross-polarization MAS (CP MAS) NMR experiments were recorded with the following parameters: RD = 7.5 or 1.5 s, contact time t_CP_ = 10 ms, 1 ms or 200 µs. For ^31^P CP MAS experiments at short RD, a ^1^H saturation step was introduced before the CP step. A two-dimensional (2D) heteronuclear ^31^P-^1^H analysis (HETCOR) was also carried out using the following parameters: RD = 1.5 s, t_CP_ = 200 µs and 160 scans were used in each 180 t_1_ increments.

### 2.4. Surface Ion Exchange Experiments

Biomimetic apatites can be exhibited on the surface of their constitutive nanocrystals a non-apatitic ionic and hydrated layer in which ions are easily and rapidly exchangeable (few seconds/minutes) [18,38]. This feature is much less observed in other previously crystallized CaP phases. Therefore, the presence of this kind of nanocrystals can be suggested indirectly by their capacity to rapidly exchange massive amounts of surrounding ions such as Mg^2+^, Sr^2+^ or CO_3_^2−^ for example. To explore the eventual presence of biomimetic apatites on the CO_2_-treated BCP surface, the cubic samples (3.3 g of each kind of BCP cube) were immersed in a Mg^2+^-containing solution (30 mL, magnesium chloride 1M (Sigma Aldrich, St. Louis, MO, USA) for 10 min. Experiments were conducted in triplicate. For each cube, three washing steps in DI water were carried out to remove all the Mg that would have not been integrated into the eventual hydrated layer. The Mg contents were titrated by atomic absorption spectroscopy (AAS, ContrAA 300, analytic Jena, Germany) after acidification in HCl 1% and filtering on a 0.2 µm syringe filter. For comparison purposes, untreated and as non-immersed BCP scaffolds were also analyzed to evaluate the possible initial Mg contents of the samples.

### 2.5. Process Parameters Influence

After studying the feasibility of the process, the potential influence of different parameters was assessed: pressure (60, 80, 100 bars), temperature (37, 50, 80 °C), time under pressure (4 h, 100 h), liquid/solid ratio (L/S = 2, L/S = 20), time of depressurization (2 min, 30 min, 24 h), resting time (0, 1, 24 h). SEM and BET measurements were carried out on each sample, as described previously. The different results, while described in the Result and Discussion part of this article, can be found in the Supplementary Information.

### 2.6. Functionalization—Copper Doping

The possibility of doping the surface of reprocessed BCP with copper ions (Cu^2+^) was studied with the aim of conveying the antibacterial properties to the ceramic samples. Three aqueous solutions of copper acetate were prepared at three different concentrations (88, 175 and 350 mg/L of copper, referred to, respectively, as *Cu Min*, *Cu Int* and *Cu Max*), and used instead of the pure deionized water to immerse the BCP scaffold inside the CO_2_ reactor. The same process (pressure, time under pressure, degassing time, rest in the aqueous environment and drying conditions) was then executed as for the “simple” ceramic modification.

Samples were then analyzed following the previously described method. In addition, EDX analyses coupled to SEM visualizations as well as ICP titrations (ULTIMA2R, HORIBA-JOBIN YVON, Kyoto, Japan) were carried out to evaluate the amount of copper that was incorporated. A release study was carried out in a slightly undersaturated Simulated Body Fluid (SBF 0.9×) to approach in vivo conditions while avoiding uncontrolled secondary precipitation that could complicate the data processing.

### 2.7. Biological Tests

Antibacterial tests were carried out on two types of bacteria (*E. coli*, Gram negative and B. subtilis, Gram positive) relevant to bone infections, by contacting the bacteria with the CO_2_-modified BCP for 24 h of incubation in an adequate culture medium (BD dehydrated, Difco^®^: LB (Luria-Bertani) broth, Miller, Edinburgh, UK). Bacteria were then grown on a nutrient agar plate for 24 h and the number of colonies formed was counted and expressed in terms of colony-forming units (CFU) to assess the potential bacterial growth inhibition action operated by the samples. Results are expressed in terms of % inhibition, as calculated by Equation (1):

(1) $$\%\;\mathrm{inhibition}=\left(1-\frac{\mathrm{Colony}\;\mathrm{number}}{\mathrm{Colony}\;\mathrm{number}\;\mathrm{on}\;\mathrm{control}\;\mathrm{plate}}\right)\times 100\%\;$$

Tests were also performed on primary mouse osteoblast MC3T3-E1 cells to validate the absence of toxicity of the Cu-free and Cu-doped scaffolds. The culture medium used was Minimum Essential Media (MEM) containing 10% fetal bovine serum and 5% penicillin/streptomycin. An incubation was used for proliferation, maintained at 37 °C and under 5% CO_2_. Osteoblast cell viability was quantified at days 3 and 7 using a dedicated kit (PrestoBlue^®^ assay (Invitrogen, Carlsbad, CA, USA)) based on the modification of the absorbance of the medium. Absorbance measurements were performed at λ = 600 nm with a Tecan Infinite M1000 Pro Multilabel microplate reader (Männedorf, Switzerland).

## 3. Results and Discussion

**Figure 1:**
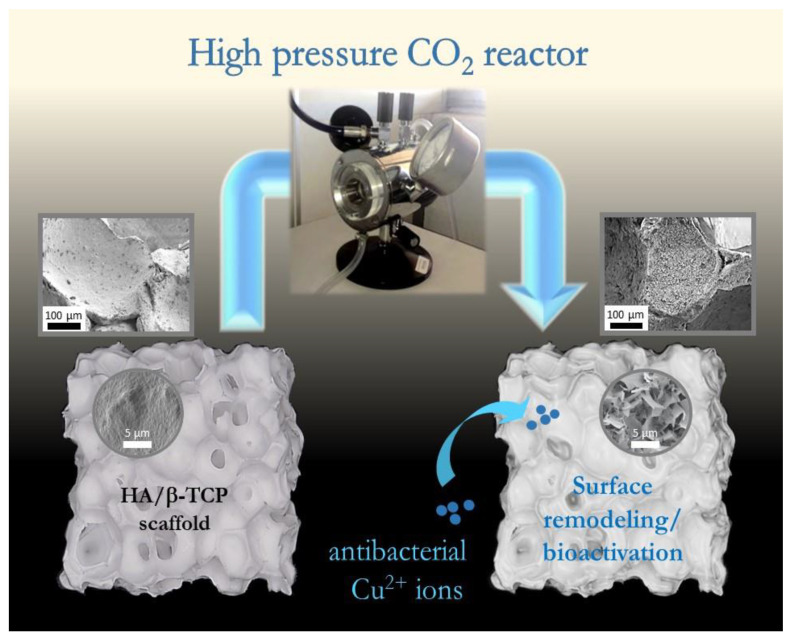
General concept of the study: a high-pressure CO_2_ reactor to be used as a tool to remodel the surface of commercial HA/β-TCP porous scaffolds, leading to high surface reactivity/bioactivity, with the potential addition of antibacterial Cu^2+^ ions.

The innovative approach of this study resides in the use of high-pressure (supercritical) CO_2_ to allow for water-immersed BCP surface remodeling via a dissolution/reprecipitation mechanism (Figure 1). In simple terms, this may be seen as a “*coating-from*” rather than a “*coating-to*” approach, by the fact that no addition of external solid substance is needed, conversely to traditional coating strategies [23,25,26,39].

The general concept of the study is based on the idea that both constituents of BCPs, namely, HA and β-TCP, become soluble in acidic conditions, making the solution pH a powerful tool for controlling CaP phases dissolution and precipitation. In addition to its already approved use at the industrial scale, including in the biomedical field [37], the choice of CO_2_ is correlated to its formation in solution of HCO_3_^−^ and CO_3_^2−^ ions, thus generating an acidification effect through the following scheme:

(2) $${\mathrm{CO}}_{2}+H_{2}O\to H_{2}{\mathrm{CO}}_{3\;}$$

(3) $$H_{2}{\mathrm{CO}}_{3}+H_{2}O\to {\mathrm{HCO}}_{3}^{-}+H_{3}O^{+}$$

(4) $${\mathrm{HCO}}_{3}^{-}+H_{2}O\to {\mathrm{CO}}_{3}^{2-}+H_{3}O^{+}$$

**Figure 2:**
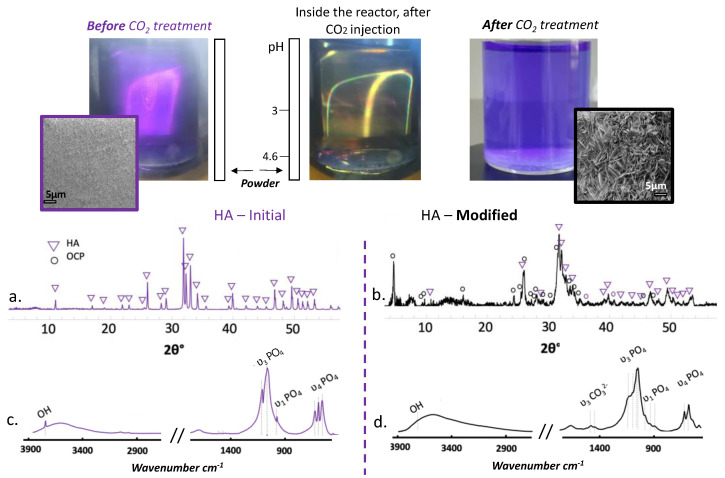
Upper part: Pictures of HA powder immersed in water in the CO_2_ reactor in the presence of bromophenol blue, before, during and after CO_2_ treatment: color change operates in the pH range 3 (yellow)–4.6 (purple). Lower part: XRD patterns (**a**,**b**) and FTIR spectra (**c**,**d**) on the initial and CO_2_-modified HA powder, respectively.

The degree of acidification is linked to the solubility of CO_2_ in water, itself related to the working temperature and pressure of the cell [40]. In the conditions used in this work (37 °C, 80 bar), a theoretical minimal pH of 3.2 may be expected, which is in the right pH domain to allow for both HA and β-TCP dissolution [41]. In a preliminary work [42], we verified that a “simple” HA powder, i.e., the most insoluble of the two phases and easily characterized by FTIR and XRD, underwent a progressive dissolution in these conditions. Figure 2 shows how the surface morphology of HA particles (powder) is modified upon such CO_2_ treatment and also shows, thanks to the addition of bromophenol blue in the medium, the clear acidification of the solution under treatment. The formation of plate-like features is a clear indication of the chemical remodeling of the surface of the HA particles. XRD analyses (Figure 2a,b) showed the presence of OCP and FTIR spectral data (Figure 2c,d) indicated the presence of carbonates, pointing to the co-presence of a carbonated apatite phase (since OCP is not known to be able to accommodate easily carbonate species). These results could be an indicator of the mechanism at stake while modifying the BCP.

We may remark that the low temperature of 37 °C is not only relevant to reaching the supercritical state of CO_2_ into the chamber’s atmosphere, but also in view of biomedical applications (physiological temperature) and allows for the preservation of metastable CaPs-targeted phases such as biomimetic apatite or lower-Ca/P precursors like OCP [38,43]. Upon depressurization of the reactor, the progressive degassing of dissolved CO_2_ is expected to induce a gradual increase in the pH until it reverted to neutrality. Therefore, the whole process can be seen as being composed of two essential steps: (i) CO_2_ pressurization of the cell, allowing for a pH drop and CaP partial dissolution and (ii) depressurization of the chamber, allowing for a pH rise and reprecipitation of metastable CaP phases (indeed, at temperatures as low as 37 °C, neither stoichiometric HA nor β-TCP can reform). It may be noted that, in case of the dissolution of HA/β-TCP phases, the release of PO_4_^3−^ and OH^−^ ions in the medium also plays a role in the rise in pH due to their propensity to combine with protons.

In comparison to CaP powders, 3D BCP scaffolds exhibit a lower specific surface area and a cohesive character with an interconnected porous network. The possibility of modifying the surface of BCP porous scaffolds thanks to this high-pressure CO_2_ process thus needs specific attention compared to treating powders, and has been evaluated here using different methods.

(i) Characterization of BCP modified by supercritical CO_2_

**Figure 3:**
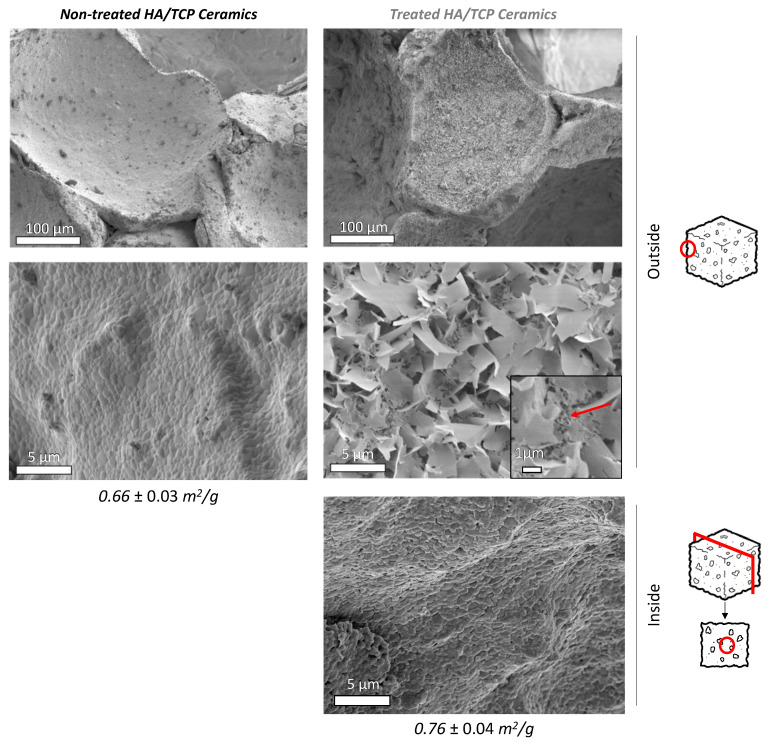
SEM pictures of non-treated and treated (4 h, 37 °C, 80 bar pressure, L/S = 2) HA/β-TCP ceramic scaffolds and corresponding specific surface area (Krypton BET). Smaller particle sizes around 0.5 µm may be seen on the outer part, with deeper porosity (red arrow).

As shown above, SEM is a particularly suited to detecting the presence or absence of surface chemical remodeling. Figure 3 shows typical surface features of both initial and CO_2_-treated BCP scaffolds (37 °C, 80 bar, 4 h). A clear remodeling of the surface of the initially smooth scaffold pores walls was achieved upon treatment. This is evidenced by the formation of plate-like particles covering the surface, with typical dimensions of around 1–5 µm on the external part of the 3 mm diameter BCP cubes and with smaller particle sizes of around 0.5 µm in the deeper porosity (red arrow on Figure 3). Despite this change in particle dimensions across the scaffold depth, the whole open porous structure appeared to be successfully remodeled. It may be noted that the (sub)micron scale of this modification throughout the outside scaffold surface (Figure 3) is well below the mean size of live cells (>10 µm), thus providing them with a renewed “covered” surface state for cell activity after implantation.

**Figure 4:**
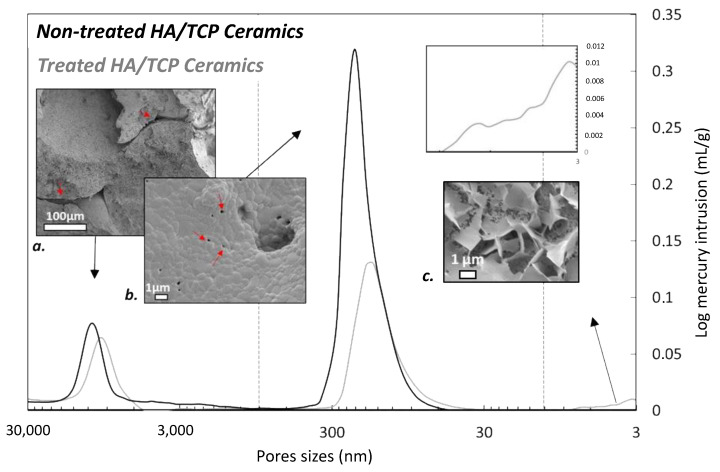
Mercury intrusion porograms of untreated (**a**,**b**) and CO_2_-treated (**c**) BCP ceramic scaffold and related SEM micrographs. Red arrows point to sintering defects in the BCP initial structure.

BET measurements showed a reproducible light increase in the specific surface area of the porous BCP cubes after high-pressure CO_2_ treatment, from 0.66 ± 0.03 to 0.76 ± 0.04 m^2^/g, thus allowing for us to expect a more extended contact surface with the surrounding medium. Mercury intrusion porosimetry analyses were carried out to analyze the modifications to the porous network in more detail (Figure 4). Two distinct pore populations are visible on initial BCP scaffolds. The first one, centered at around 10.5 µm, could match the gaps between pores, as shown in Figure 4a. The second (most prominent) pore population is observed close to 200 nm, itself composed of several subpopulations, as indicated from the analysis of the derivative function, which shows several inflexion points in this area (Supplementary Materials, Figure S1). This could be due to the sintering defects (red arrow, Figure 4a,b). In contrast, several modifications are seen for the treated BCP. The two main populations that were previously observed on untreated BCP are downshifted toward slightly smaller pore sizes, of around 10.5 µm and 150 nm, respectively. This slight decrease in main pore diameters is indicative of the surface remodeling observed by SEM and unveiling of newly precipitated phases/crystals covering the surface, thus generating a decreasing tendency for the original pore diameters. In addition, a new mesoporosity was observed on the treated scaffold, with nanometer-sized pores (~3–9 nm). This new nanometer-scale porosity could be due to the network formed by the newly precipitated entangled platelets detected by SEM (Figure 4c). The occurrence of nanosized pores has already been reported in biomimetic apatites and attributed to the 3D organization of apatite nanocrystals [25].

These cumulated data, arising from SEM, BET and Hg-porosimetry, demonstrate that BCPs subjected to this high-pressure CO_2_ process undergo a surface remodeling with newly formed crystals, whose morphology, size and 3D organization are close to those reported for biomimetic nanocrystalline apatites. To shed more light on the nature of the formed precipitates, XRD and FTIR analyses were carried out (Figure S2a,b). However, the data obtained via these bulk characterization techniques did not allow for the detection of substantial new phases. Nonetheless, a decrease of the HA/β-TCP weight ratio from 70/30 (experimental starting values) to 85/15 was evaluated (Figure S2c, estimated error of 5% on mass% assignments), pointing to a significant loss of β-TCP, the most soluble of the two phases in the conditions of the process. Contrary to the powders, BCP scaffolds exhibit a low surface area and an overall mechanical cohesion that limits the extent of the chemical alterations generated by the CO_2_ treatment to only the surface of the scaffold’s open porosity. This fits well with our goals aiming at remodeling only the surface of BCPs while preserving their general integrity and porous character, in view of subsequent uses in previously identified bone applications. Nonetheless, in these conditions, neither XRD nor FTIR offers enough resolution to access the top surface modifications generated here. Therefore, more advanced analyses were performed with less conventional approaches, such as solid-state NMR. Indeed, NMR proved to be a particularly suitable method for characterizing bone mineral and CaP phases [43,44,45,46,47,48,49,50,51], and presents the advantage of allowing for biomimetic apatites to be distinguished from OCP that otherwise exhibits rather close features using XRD and FTIR [43].

**Figure 5:**
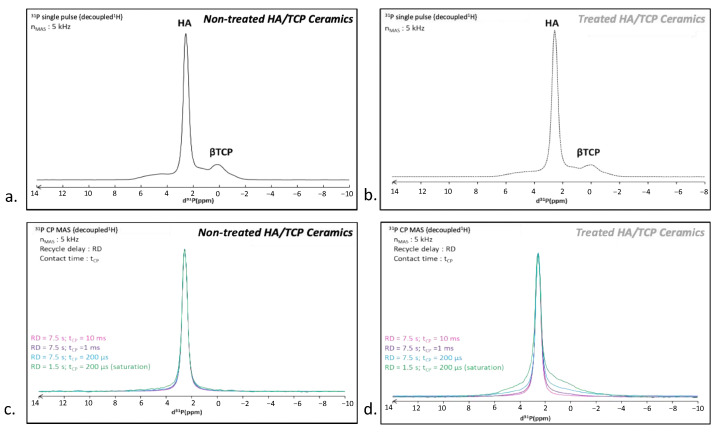
Single-pulse ^31^P MAS NMR spectra of (**a**) untreated and (**b**) CO_2_-treated BCP scaffold. ^1^H-^31^P CP MAS NMR spectra of (**c**) untreated and (**d**) CO_2_ treated BCP scaffold at various conditions indicated on the figure. Arrow in (**d**) indicates the surface species induced by the CO_2_ treatment.

A ^31^P single pulse MAS NMR spectrum was recorded on the untreated BCP scaffold (Figure 5a). Two main signals were detected, at 2.6 and a broad one spanning from 6 to 0 ppm. The most intense 2.6 ppm peak can be assigned to the HA component [45] while the 6–0 ppm broad signal corresponds to the β-TCP phase [46,47], in agreement with the initial composition of these biphasic scaffolds. These two components were also detected on the CO_2_-treated BCP without major visible alteration (Figure 5b). In order to enhance the response of protonated phases, additional measurements were made in ^1^H → ^31^P cross polarization mode. Note that, in this configuration, the β-TCP phase (exempt of protons) cannot be visualized. Several measurements were made by decreasing the contact time (from 10 ms down to 200 µs), allowing for us to probe the nearest environment of phosphorus nuclei. As expected, the ^1^H-^31^P CP MAS NMR spectrum of the untreated scaffold displayed the single 2.6 ppm peak characteristic of HA [45], whatever the contact time (Figure 5c). In contrast, the CO_2_-treated BCP behaved differently (Figure 5d) as the ^1^H-^31^P CP MAS NMR spectrum revealed a wide halo at the peak basis, which increases in intensity as the contact times decreases. A shortened recycling delay (RD) (from 7.5 s down to 1.5 s) made this large signal even more obvious. Through comparison with the untreated sample, we assigned this broad resonance to surface phosphate species induced by the CO_2_ treatment. The enlargement is indicative of a distribution of chemical shifts and, thus, the presence of disordered phase(s) at the surface involving strong ^1^H-^31^P interactions at short range, thus pointing to the presence of phase(s) rich in H_2_O and/or HPO_4_^2−^ ions. This is characteristic of crystallographic disorder and/or of a high degree of hydration compared to HA [42,48].

**Figure 6:**
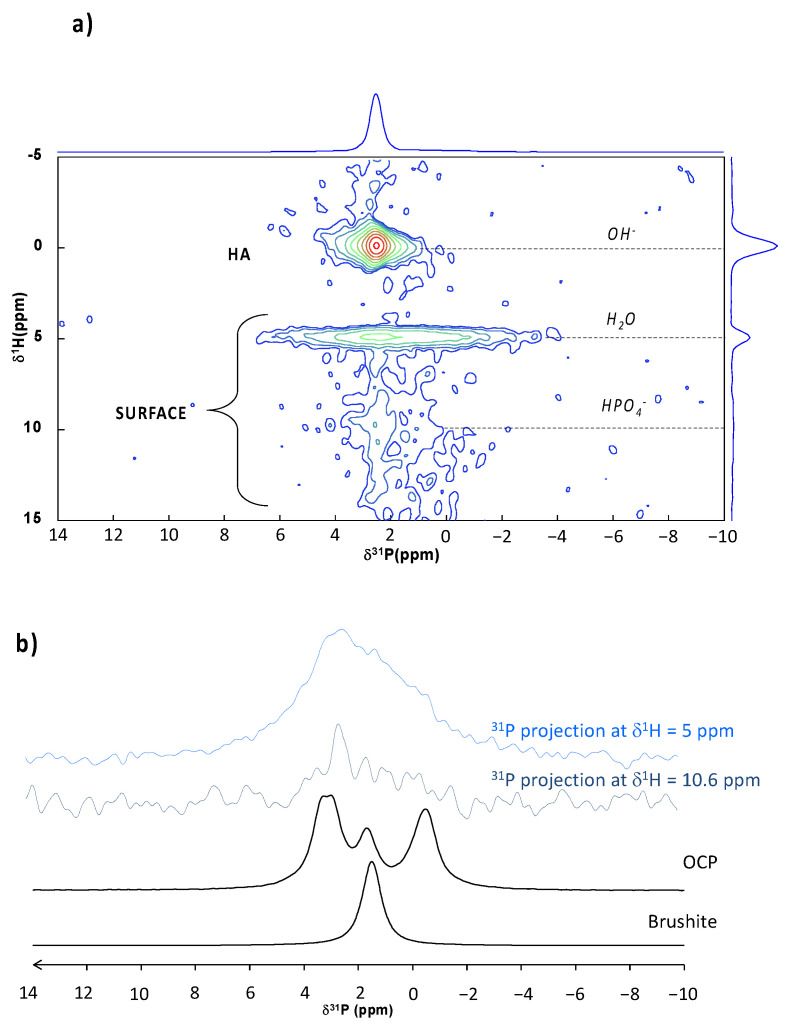
(**a**) ^1^H-^31^P 2D HETCOR NMR spectrum of CO_2_ treated BCP scaffold. (**b**) Comparison of 31P signal from surface species (projections from the 2D HETCOR spectrum) with ^31^P NMR spectrum of reference CaP samples.

To try to further identify the disordered CaP phase(s) composing the BCP remodeled surface, a 2D ^1^H-^31^P HETCOR experiments was performed (Figure 6a). This analysis aims to provide information on the proximity and interaction between ^31^P and ^1^H nuclei. Two distinct correlation zones can be identified. A first correlation (denoted as HA in Figure 6a) shows the interaction between a rather narrow correlation between ^31^P and protons at 0 ppm, which is characteristic of PO_4_^3−^/OH^−^ interactions within the HA phase [43,44,45]. In addition, other correlations can also be seen (denoted SURFACE in Figure 6a) between the broad ^31^P signal from surface species and ^1^H arising from H^2^O molecules (δ_1_H = 5 ppm) but also, to a lesser extent, from HPO_4_^2−^ ions (centered at δ^1^H = 10.6 ppm), confirming the presence of at least one hydrated phase including some hydrogen phosphate ions after CO_2_ treatment. The ^31^P contributions (projections from the 2D HETCOR spectrum) from phosphate surface species have been plotted in Figure 6b to better visualize the underlying contributions, compared with the reference spectra of OCP and DCPD (internal collection). Thus, despite the presence of H_2_O and HPO_4_^2−^, the presence of DCPD—which exhibits a highly ordered structure—at the surface of the treated samples can be ruled out (or is present in small amount). On the other hand, we note that the ^31^P surface signal spanned same range as the OCP reference spectrum and correlated with H_2_O and HPO_4_^2−^ species similar to OCP. Thus, this analysis might suggest the presence of a poorly crystallized OCP-like phase (e.g., in the process of crystallization) at the surface of the treated scaffold. These findings are in coherence with previously reported data of HA and ACP powders treated with the same process [42]. However, if the co-presence of biomimetic apatite was clearly identified in this previous work using powders, its presence on the treated BCP scaffold cannot be asserted here on the sole basis of solid-state NMR. Indeed, since the BCP bulk is mainly composed of HA, an eventual trace of biomimetic apatite at the surface could not be detected in these conditions due to the superposition of the corresponding signals.

**Figure 7:**
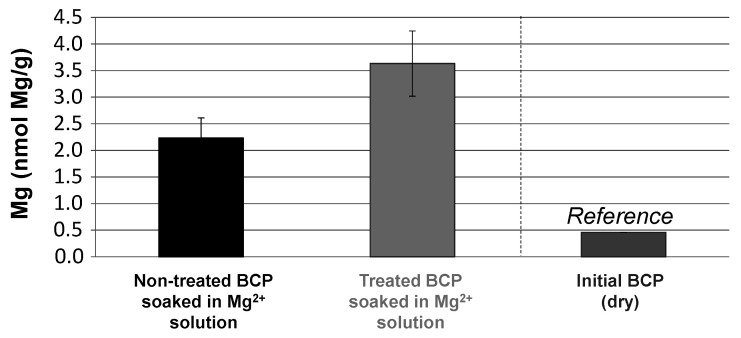
Average Mg^2+^ ion content of sample (BCP cubes) treated or not with high pressure CO_2_ (standard protocol) and after immersion in a solution of Mg^2+^ ions (10 min, magnesium chloride 1M). The initial Mg content of these commercial ceramics (far right) was added for comparative purposes.

To test the co-presence of biomimetic apatite on the CO_2_-treated BCP remodeled surface, fast Mg^2+^ → Ca^2+^ ion exchange experiments were carried out in solution. Indeed, one feature of bone-like biomimetic apatites is their very high surface ion mobility allowing for fast exchanges (within few seconds/minutes) with various types of ions contained in the surrounding aqueous medium. This property is naturally used by bone mineral in vivo to play an active role in homeostasis [52]. Such fast ion exchanges were, for instance, reported in the example of the replacement of surface Ca^2+^ ions (contained in the hydrated layer on biomimetic apatite nanocrystals) by Mg^2+^ or Sr^2+^ ions [18]. This approach may be seen as an indirect way of testing presence of biomimetic apatite, since OCP or DCPD are not known to share this property. Indeed, although ion-doped OCP [53,54] and DCPD [55,56] have been reported, this necessitates directly adding the doping ions during synthesis. Treated and untreated BCP scaffolds were, thus, immersed in a Mg^2+^-containing solution (10 min, magnesium chloride 1M) followed by thorough washing with DI water, and the Mg contents were titrated by AAS. Results are reported in Figure 7 (expressed per gram of ceramic). Titration on raw BCPs indicates an initial Mg content close to 0.5 nmol Mg/g, with magnesium being a frequent impurity in other alkaline-earth sources, such as calcium ores. The Mg content on CO_2_-untreated BCP cubes simply immersed in the Mg^2+^ solution led to an Mg content of 2.2 ± 0.4 nmol/g, which is presumably assignable to retention by the HA phase present in the BCP composition. Although some ion sorption ability of HA is significantly lower than that for biomimetic apatites, the possibility of HA capturing ions to some extent has been reported, especially for the remediation of polluting cations [57,58]. It is then interesting to compare this Mg content value to the CO_2_-treated BCP scaffolds, which was found to be noticeably higher (by a factor 1.6) and reaching 3.6 ± 0.6 nmol Mg/g. The CO_2_ treatment thus allowed an additional Mg incorporation of 1.4 ± 0.7 nmol/g. This important increase is in accordance with our hypothesis of the presence of a biomimetic apatite phase besides OCP (a precursor of biomimetic apatite formation) in the treated sample, which is coherent with the NMR results. These conclusions are also similar to those obtained in our preliminary study on the modification of HA and ACP powders by high-pressure CO_2_ [42]. It is likely that the mechanism during the process leading to the precipitation of those phases is similar. Additionally, these two phases (biomimetic apatite and OCP) are known to form plate-like particles, as was observed on the new surface layer shown in Figure 3. In contrast, the presence of an amorphous CaP phase on the CO_2_-treated BCPs seems improbable from the absence of globular morphology typical of ACPs. Concerning the biomimetic apatite phase, it is logical to suspect carbonation (as in bone), considering the presence of carbonate ions in water during the high-pressure CO_2_ treatment. To further investigate this latter point, a test was carried out in the CO_2_ cell on an “initially non-carbonated” biomimetic apatite gel, precipitated following a previously reported protocol but without final freeze-drying [38]. As shown by FTIR analyses (Figure S2c), a clear development of two vibrational bands characteristic of carbonate species in apatite was detected in the domains 800–900 cm^−1^ and 1350–1600 cm^−1^, corresponding, respectively, to the vibration modes ν^2^CO_3_ and ν^3^CO_3_ [59]. These findings thus indicate that, in the presence of high-pressure CO_2_, any nanocrystalline biomimetic apatite formed upon reprecipitation would readily get carbonated, thus approaching the features of natural bone apatite.

Considering the findings presented above, the overall surface remodeling scheme may presumably be summarized by the following main steps:

1. Partial surface dissolution of the HA: Ca_10_(PO_4_)_6_(OH)_2_ and β-TCP: Ca_3_(PO_4_)_2_ phases upon acidification under high-pressure CO_2._
2. Local generation of dissolved calcium and phosphate ions (plus OH^−^ ions recombining with H^+^ to form H_2_O), besides carbonate ions arising from dissolved CO_2._
3. Local re-increase in the pH linked to the partial dissolution of the phosphate ions present on the surface of the ceramics and during depressurization. Local supersaturation.
4. Re-precipitation of nonstoichiometric carbonated biomimetic apatite:

~ Ca_10−x_(PO_4_)_6−x_(HPO_4_,CO_3_) × (OH, ½ CO_3_)_2−x_ and OCP: Ca_8_(PO_4_)_4_(HPO_4_)_2_.5H_2_O

It should be noticed that possible precipitation occurred very locally during the process, due to the release of ionic species and the modified pH (linked to the phosphate release). Thus, several local cycles of dissolution/precipitation could occur during the CO_2_ treatment.

In this scheme, note that we hypothesized a chemical composition for biomimetic apatite that is generic and is often considered in first approximation. In actual nanocrystals, especially those with a poor crystallinity, the presence of a hydrated ionic layer on the nanocrystals surface probably leads to a departure from this generic formula in a way that is still under exploration. This formula, however, aims to highlight the nonstoichiometric character of such biomimetic apatite, which also explains its metastability and high related reactivity [60]. Note also that the OCP phase itself may depart somewhat from this chemical formula in the present study, taking into account the NMR data suggesting a concerning OCP phase, which could be in the course of crystallization or transformation into biomimetic apatite. Since the initial Ca/P ratio of the BCP surface (assuming a homogeneous initial distribution of the HA and β-TCP components on the commercial scaffolds) is lower than 1.67—it can be calculated at 1.60 for the BCP used here—then the re-crystallization of CaP phases with a lower Ca/P ratio such as biomimetic apatite and OCP (Ca/P = 1.33) is totally coherent. According to our previous experiments on powders [42] and the literature [61], it is possible that polynuclear germs could form, followed by crystal growth, corresponding to OCP formation. The HA original grains are likely more stable than β-TCP during the dissolution phenomenon, and might act as nucleation points for the newly precipitated phases. Indeed, no signs of homogeneous precipitation could be detected in the CO_2_ cell (no blurring is observed in the medium). The precipitation of biomimetic apatite might occur directly from the supersaturated medium as for OCP, but may also form in a subsequent stage, upon hydrolysis of OCP. In any case, the presence of these phases (with OCP being a known precursor in vitro and in vivo of nanocrystalline apatite) validates the concept pursued in the present work, aiming to modify the surface of commercial BCPs to expose such highly reactive CaP phases for an increased bioactivity, based on previous biological evidence from the literature [22,25].

(ii) Influence of operating parameters

At this stage, it was interesting to investigate the possible effect of the main parameters of the supercritical CO_2_ cell and experimental conditions. The first parameter investigated was the resting time of BCP after the process into the aqueous environment (set to 1 h in the reference protocol). Samples obtained without any resting time showed partial re-precipitation; therefore, nucleation starts before the beginning of the resting time, probably during the outgassing step. In contrast, samples left for a longer resting time (e.g., 24 h) showed an increased specific surface area (from 0.76 ± 0.03 to 0.98 ± 0.03 m_2_/g) and a denser surface coating as seen by SEM (Figure S3), both revealing a higher degree of conversion. Temperature (T) and pressure (P) were two other studied parameters. Upon T increase at 80 bar, the pH of water increases due to the lower solubilization of CO_2_ in that phase (as evidenced by PHREEQC calculations, Figure S4a), which leads to HA and β-TCP solubility decrease (retrograde solubility behavior of several CaP phases). Although reaction kinetics may be favored due to the T increase, these effects do not thermodynamically favor the surface remodeling of the BCP cubes of interest here, which is experimentally seen by a limited change in surface features upon T increase from 37 to 50 and 80 °C (Figure S5). Conversely, a pressure increase at 37 °C tends to facilitate CO_2_ solubilization in water (thus the related pH drop) and slightly increase HA/β-TCP solubility (Figure S4b). Indeed, experiments performed at 100 bar (rather than 80) suggest that higher pressure favors the surface remodeling, as evidenced by the occurrence of holes of about 1 µm wide, suggesting the disappearance of some initial CaP grains (Figure S6). Another relevant parameter to follow is the time spent under CO_2_ pressure. As expected, allowing for more time (e.g., 100 h rather than 4 h) for BCP in an acidic environment favors an important surface remodeling (Figure S7). The amount of water in the cell, as determined from the L/S mass ratio, also appears as a key parameter, as it is directly related to the CO_2_ dissolution and pH drop. The absence of water or a simple humidification of the BCP samples were not found to allow for BCP surface dissolution/re-precipitation. In contrast, too high a water content (e.g., L/S = 20 compared to 2 in the reference protocol) dilutes the released ions and disfavors CaP reprecipitation by limiting the local supersaturation, thus leading to less efficient remodeling (Figure S8). Finally, the depressurization speed (to get back to atmospheric pressure) regulates the kinetics of pH change, and is also likely to influence the BCP surface remodeling. Indeed, the medium pH only significantly increases toward the end of the depressurization step, at around 25 bar. Noticeable signs of surface remodeling were effectively seen by modulating the duration of the depressurization step from 2 min to 24 h (Figure S9).

(iii) Functionalization

Beyond this point, we can also propose further widening this concept by exploiting the high reactivity of biomimetic apatite, by adding some biologically active ions to the supercritical CO_2_ cell ions. Indeed, such ions could then be incorporated during the reprecipitation step upon depressurization (step #4 in the above scheme), thus allowing for new functionalities to be adjoined to the scaffolds, such as antibacterial properties.

In this study, we investigated this point by dissolving copper acetate in the solution containing the BCP scaffolds to be treated in supercritical CO_2_. Copper ions indeed proved to be very relevant in the biomedical field and, in particular, for bone engineering thanks to their antibacterial and pro-angiogenic properties, as well as for potentially stimulating osteoblast cell activity [33,34]. Cu^2+^ ions have also shown an ability to integrate, at least to a certain extent, the apatite lattice, typically in replacement of calcium ions [62,63]. Being able to incorporate copper into the structure during our “coating-from” process could be highly relevant to add bioactivity to the BCP “CO_2_-activated” scaffolds. Indeed, integration of this element into the scaffold could be of interest to allow for the in vivo release of the antibacterial/bioactive Cu^2+^ ions after implantation and/or upon resorption.

In order to evaluate the amount of copper (Cu^2+^) integrated into the scaffold after the process, ICP measurements were carried out on the dissolved solids, and also by titration of the medium before and after CO_2_ treatment (the incorporated copper content being then determined from the difference between these two values). The two sets of data proved to be in agreement with each other. Tests were carried out for three copper acetate concentrations, denoted *Cu Min*, *Cu Int* and *Cu Max,* as described in the experimental section. ICP results led to the doping rates reported in Table 1.

It is important to note, however, that the introduction of a copper salt (here copper acetate) to the high-pressure cell before CO_2_ treatment may impact the pH value (initial but also along the process), depending on its concentration. As it is not possible to directly measure the pH inside the reactor during the process because of the high pressure, the pH at CO_2_ saturation was estimated using the PHREEQC software (version 2) for the three different initial copper concentrations studied here (Figure S10). As may be seen, upon Cu increases in the medium, the pH at saturation tends to become less acidic, negatively influencing the BCP surface remodeling. Indeed, only very partial dissolution/re-precipitation seems to have occurred in the *Cu Max* condition. In contrast, as expected, for the lower copper concentrations *Cu Int* and *Cu Min*, surface remodeling was clearly visible by SEM (Figure S11)—although full surface coverage was not reached in these tested conditions. Hg porosimetry also showed a significant downshift of the 210 nm pore size peak to 162 nm, thus illustrating the effective surface remodeling of the BCP porous scaffolds.

We may also notice that, for the highest concentration of *Cu Max*, at least one new phase was formed, as seen by SEM observations (Figure S11), in the form of flower-like structures. EDX analyses revealed that they were mainly composed of copper and phosphorus (Figure S12), suggesting the possible additional formation of copper phosphate(s) Cu_3_(PO_4_)_2_. nH_2_O.

**Figure 8:**
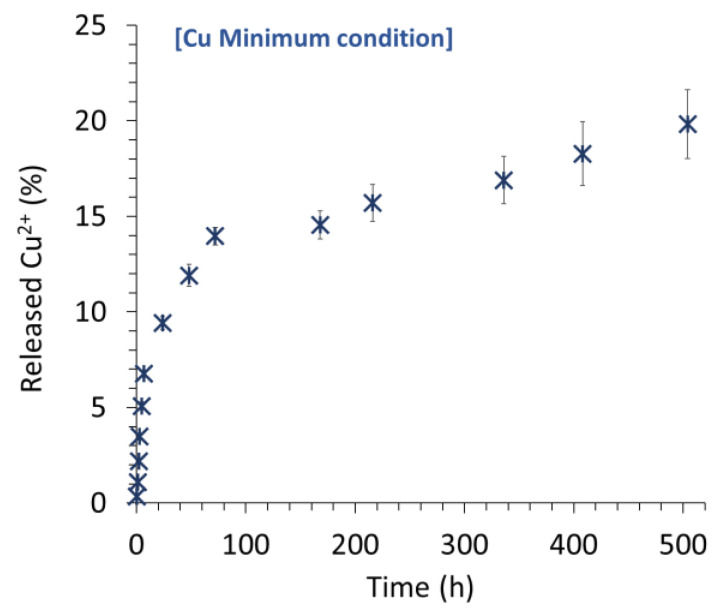
Copper release in SBF 0.9× over 3 weeks, example of the *Cu Min* initial concentration.

The Cu^2+^ release profile of Cu-containing samples was explored in SBF 0.9x (e.g., Figure 8 for the example of *Cu Min*). Several trends may be identified: a first significant “burst release” effect occurred in the first hours, followed by a decrease in the release kinetics until a rather steady plateau was reached. It may be noted that up to 20% of the copper ions included in the treated BCPs were released after three weeks in these cell-free conditions. These different release stages might reflect the different types of incorporation of copper ions in the modified BCPs, e.g., within the formed apatite nanocrystals (whether from the hydrated layer or the apatitic bulk), within the OCP, or in Cu-containing secondary phase such as copper phosphate(s) that may be more or less present as a function of the copper concentration. The clear identification of such phases is, however, quite delicate considering their top-surface localization, leading to characterization difficulties. Additionally, these “chemical” considerations must contain mechanisms of diffusion of released ionic species within the porous network of the scaffolds. However, these findings confirm the possibility of loading and further releasing copper ions in view of local action (e.g., antibacterial, pro-angiogenic or as osteoblast activator).

**Figure 9:**
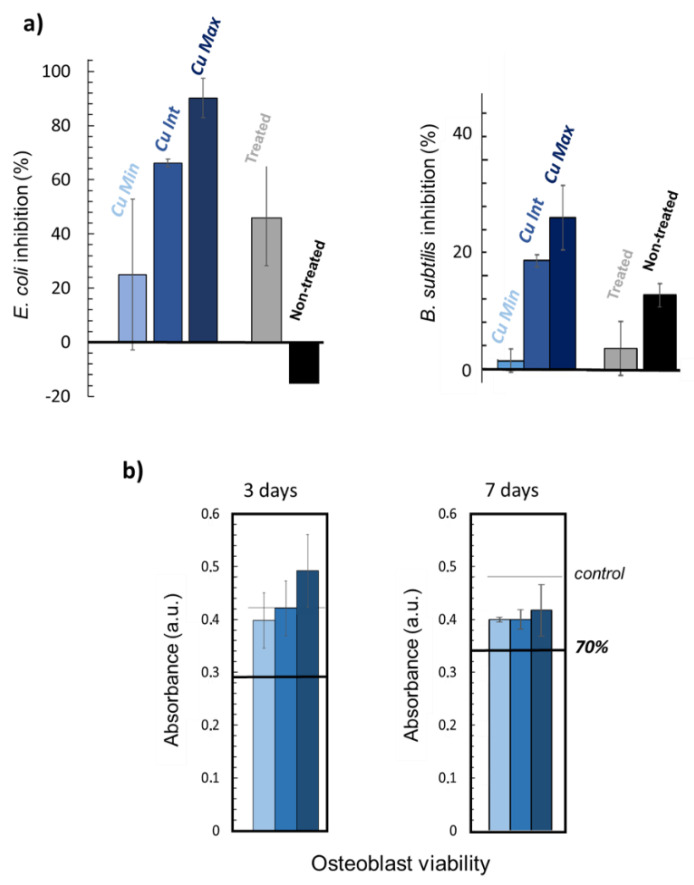
In vitro evaluations of Cu-doped samples: (**a**) Antibacterial effect on *E. coli* and *B. subtilis* bacteria, (**b**) Osteoblast viability at days 3 and 7. The dark line represents the values corresponding to 70% cell viability.

Biological tests were then carried out on the three types of scaffolds (*Cu Min*, *Cu Int* and *Cu Max*). First, antibacterial tests showed an inhibitory effect of the simply modified scaffold without any supplementary ions on both *E. coli* and *B. subtilis* bacteria (Figure 9a). This effect is likely related to the modification of the surface topography of the BCPs upon dissolution/reprecipitation, affecting bacteria survival/spreading. Copper-loaded samples demonstrated a greater inhibitory effect upon increasing the Cu content for both types of bacteria (although more markedly for the Gram negative *E. coli* strain). This antibacterial effect could be due to a destabilization of the membrane, as already observed with copper ions [64,65]. These results thus confirm that doping with antimicrobial ions such as Cu^2+^ during the CO_2_ treatment process allows for additional properties to be conferred to the treated scaffolds, allowing for tailorable functionalized bone substitutes to be envisioned.

Finally, viability tests were performed on osteoblast cells contacted with the copper-loaded scaffolds (Figure 9b). Although the viability at day 3 appears to be somewhat more related to the copper concentration than at day 7, this effect is not statistically significant when considering the larger error bars. At day 3, osteoblast viability is overall very satisfactory and close to the control (represented by the black line). At day 7, a slight decrease in viability can be noted compared to the control, but the samples do not exhibit a high cytotoxicity in any case.

## 4. Conclusions and Remarks

This study aimed to explore the concept of BCP surface remodeling via an original approach based on the use of a supercritical CO_2_ and an aqueous environment, which is propitious to allow for the dissolution–reprecipitation phenomena by controlling the media pH media thanks to pressure and temperature. We validated this concept based on a combination of techniques that showed the formation of plate-like structures (SEM) on the CO_2_-treated BCP porous surfaces. These structures appear to be composed of bioactive OCP and (carbonated) biomimetic apatite, as concluded from a set of converging methods including solid-state NMR, ion exchanges and FTIR. However, OCP is a precursor for biomimetic apatite and will transform into this after implantation by hydrolysis [66,67]. In addition to the nanocrystalline apatite that builds up natural bone, OCP has also been reported as bioactive and favorable for bone regrowth [68]. The remodeled surface exhibits a larger surface area and additional nanometer-scale pores, which could prove to favor protein adsorption and subsequent cell activity. This process may thus be viewed as an original “coating-from” rather than the regular “coating-to” approach. It is easy to put in place, using an industrializable CO_2_ methodology, and can boost the bioactivity of existing, commercial BCPs, thus providing an innovative way of renewing their relevance in the bone engineering field. Additionally, this approach could be extended to other 2D or 3D bioceramics, e.g., those obtained by additive manufacturing.

## Figures and Tables

**Table 1:** Copper concentration (µg Cu/g of ceramic) in *Cu Min*, *Int* and *Max* samples, determined by ICP.

	Copper Concentration (µg Cu/g of Ceramic)
*Cu Min*	157.17 ± 3.73
*Cu Int*	346.52 ± 9.61
*Cu Max*	563.51 ± 8.98

## Data Availability

Data can be provided on demand by contacting the corresponding author.

## Supplementary Materials

The following supporting information can be downloaded at: https://www.mdpi.com/article/10.3390/ma15207306/s1, Figure S1: First and second derivative functions of porogram for untreated and treated BCP scaffolds; Figure S2: (a) XRD pattern and (b) FTIR spectrum for modified and initial scaffolds. (c) Mass% of modified or initial scaffold calculated thanks to Match^®^ Software and RIR methods from XRD data (evaluated relative error: 5% on mass% assignment). (d) Carbonation effect upon treatment in the CO_2_ high pressure reactor of a non-carbonated nanocrystalline apatite gel; Figure S3: SEM observations on the effect of resting time in the CO_2_ process; Figure S4: PHREEQC calculations: evolution of pH, HA and β-TCP solubility versus temperature at 80 bar (a) and pressure at 37 °C (b); Figure S5: SEM observations on the effect of temperature in the CO_2_ process at 80 bar; Figure S6: SEM observations on the effect of pressure in the CO_2_ process; Figure S7: SEM observations on the effect of time under pressure in the CO_2_ process; Figure S8: SEM observations on the effect of the L/S ratio in the CO_2_ process; Figure S9: SEM observations on the effect of the depressurization (outgassing) time in the CO_2_ process; Figure S10: PHREEQC calculations: effect of Cu^2+^ content on the pH at CO_2_ saturation at 37 °C and 80 bar pressure; Figure S11: SEM observation of CO_2_-treated scaffold with different concentrations of copper; Figure S12: EDX analyses on “*Cu Max*” modified scaffolds.
