# Supplementary material for: Bio-Activation of HA/β-TCP Porous Scaffolds by High-Pressure CO2 Surface Remodeling: A Novel “Coating-from” Approach
Source: Materials (Basel). 2022 Oct 19;15(20):7306. doi: 10.3390/ma15207306 (PMC9610974; doi:10.3390/ma15207306)
Supplement: Supplementary file 1 [file materials-15-07306-s001.zip › materials-1951360-supplementary.pdf]

Supplementary Information

Figure S1: First and second derivative functions of porogram for untreated and treated BCP scaffolds. Derivative functions show the existence of several inflexion points (= pore populations). These data highlight a redistribution of the pores due to the CO_2 treatment in addition to a decrease of the pore volume. In particular, the population at ~212 nm is no longer present on the modified samples. Also, a pore population shift is visible, with the maximum passing from 150 down to 138 nm. In addition, new pores appear at 85 nm on the treated scaffolds.

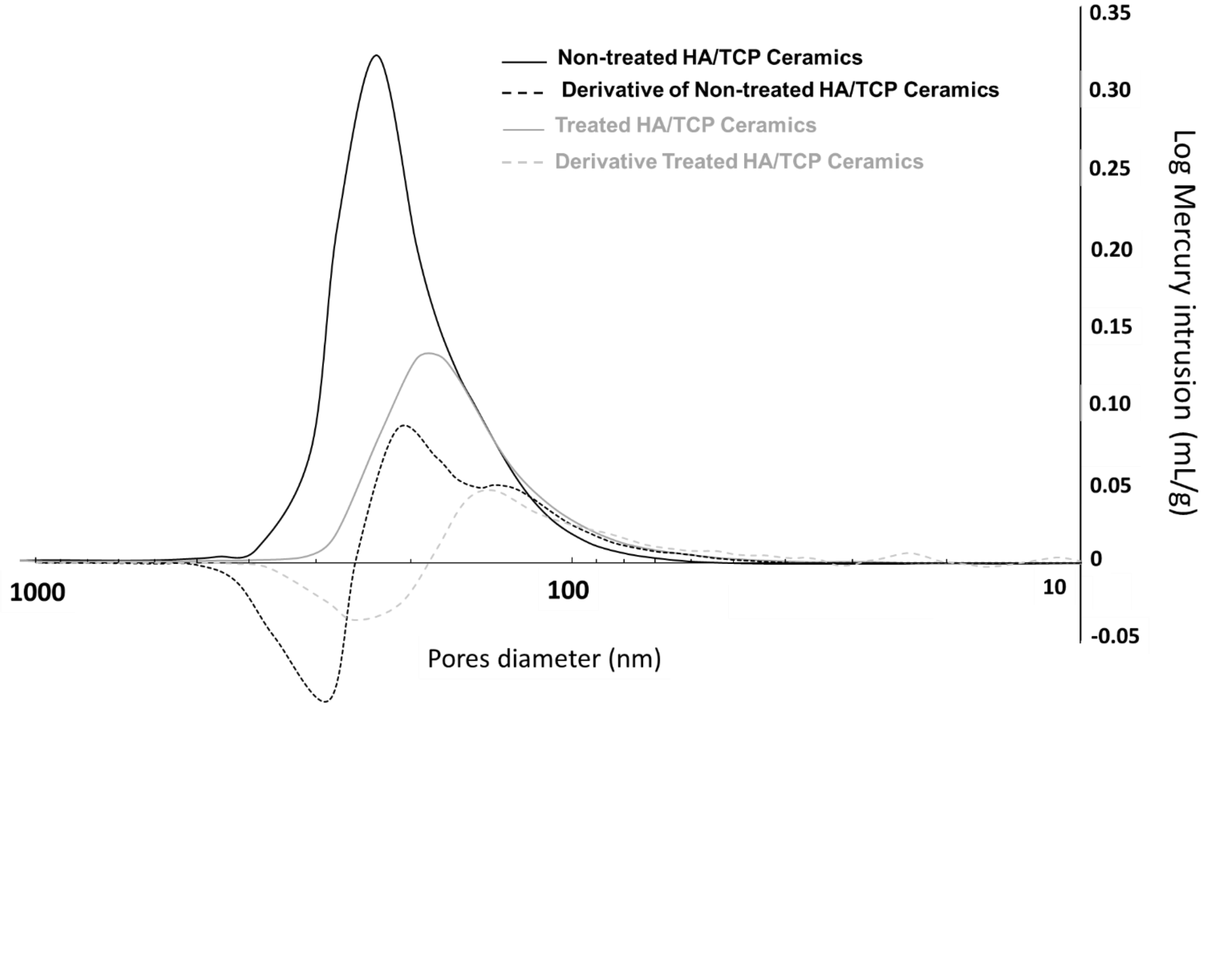

Figure S2: a) XRD pattern and b) FTIR spectrum for modified and initial scaffolds. c) Mass % of modified or initial scaffold calculated thanks to Match® Software and RIR methods from XRD data (evaluated relative error: 5% on mass % assignment). d) Carbonation effect upon treatment in the CO_2 high pressure reactor of a non-carbonated nanocrystalline apatite gel.

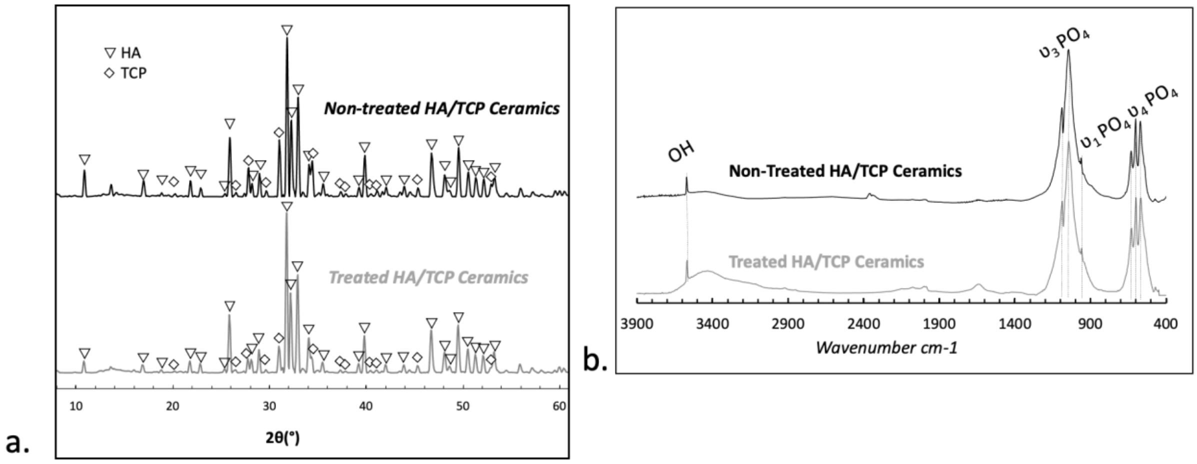

	Mass%	
	HA	βTCP
Theoritical **Non-Treated HA/TCP Ceramics**	65	35
XRD Measured **Non-Treated HA/TCP Ceramics**	70	30
XRD Measured Treated HA/TCP Ceramics	85	15

c.

d. → next page

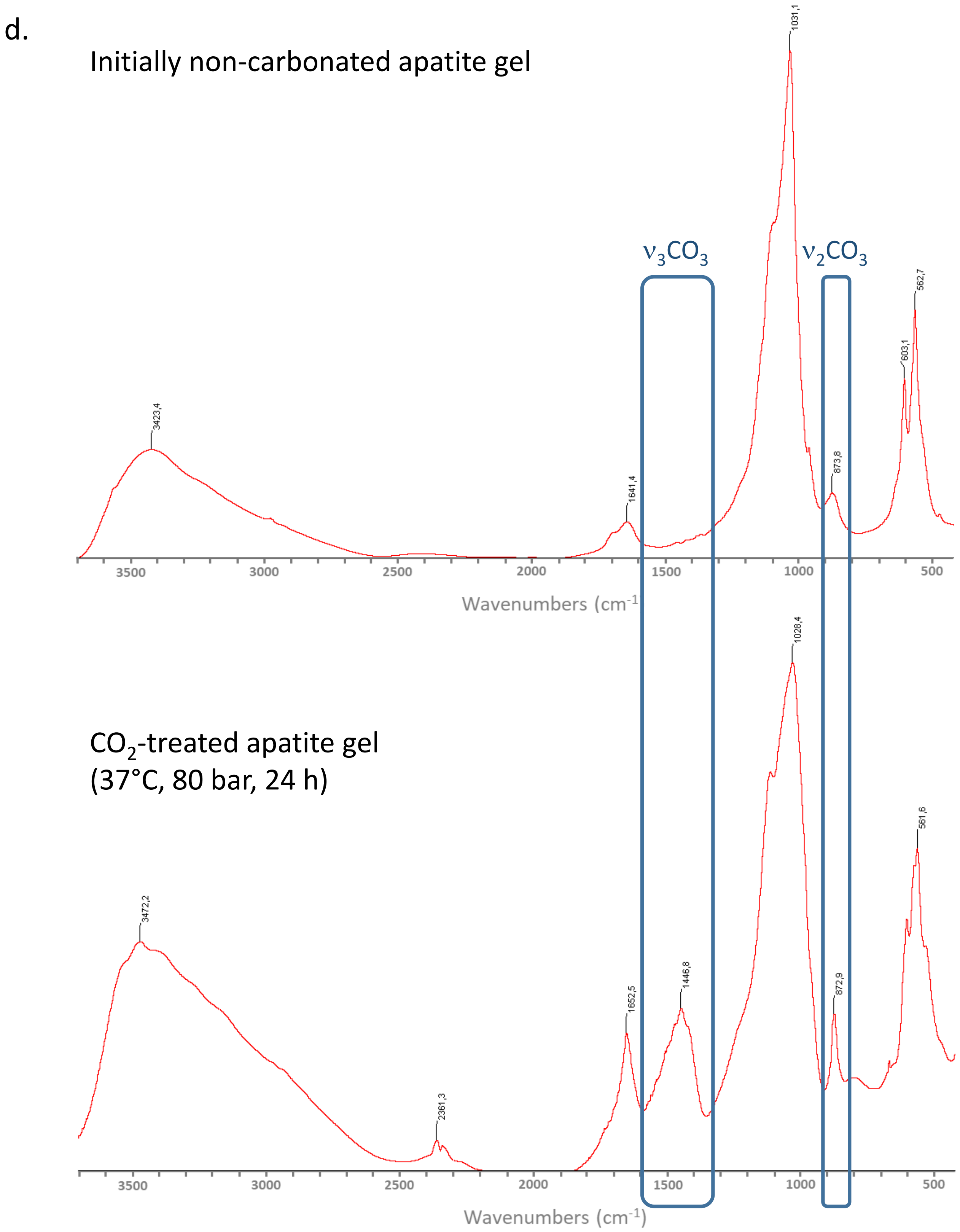
d.
Initially non-carbonated apatite gel
ν3CO3
ν2CO3
1031,1
562,7
603,1
3423,4
1641,4
873,8
3500
3000
2500
2000
1500
1000
500
Wavenumbers (cm-1)
CO2-treated apatite gel
(37°C, 80 bar, 24 h)
1028,4
561,6
3472,2
1446,8
1652,5
872,9
2361,3
3500
3000
2500
2000
1500
1000
500
Wavenumbers (cm-1)

Figure S3: SEM observations on the effect of resting time in the CO_2 process

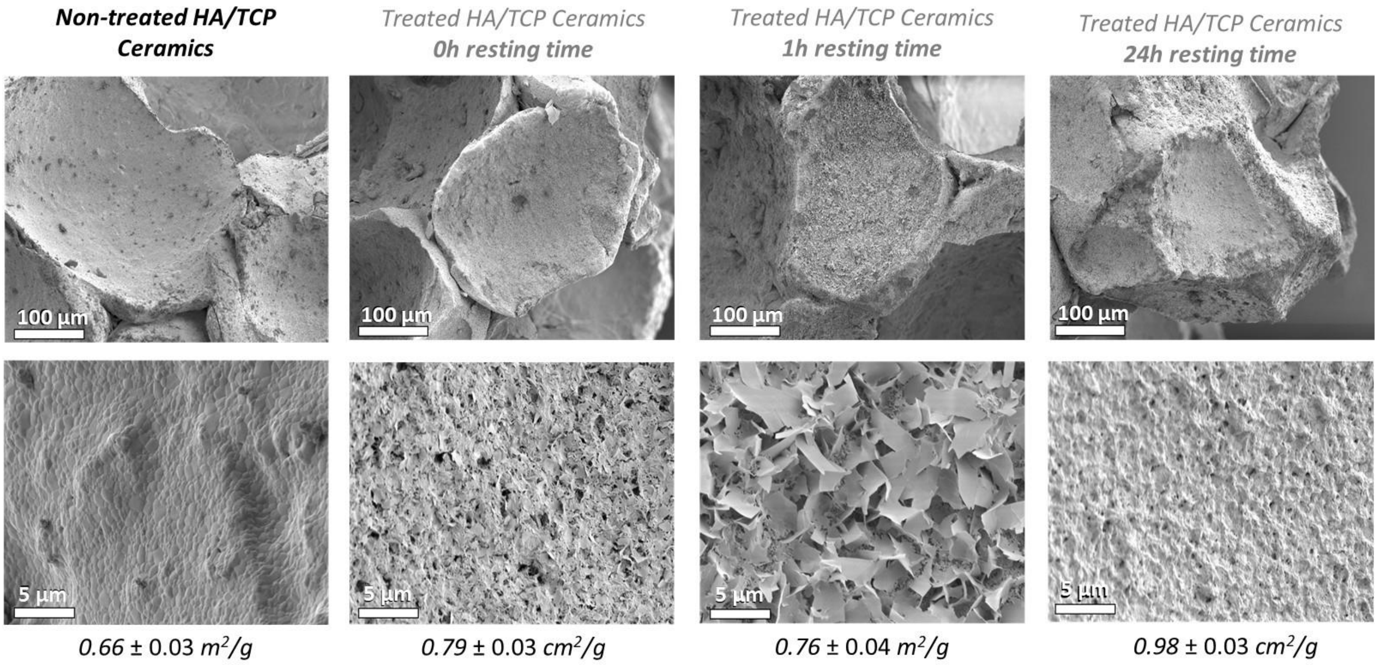

Figure S4: PHREEQC calculations: evolution of pH, HA and β-TCP solubility versus temperature at 80 bar (a) and pressure at 37°C (b)

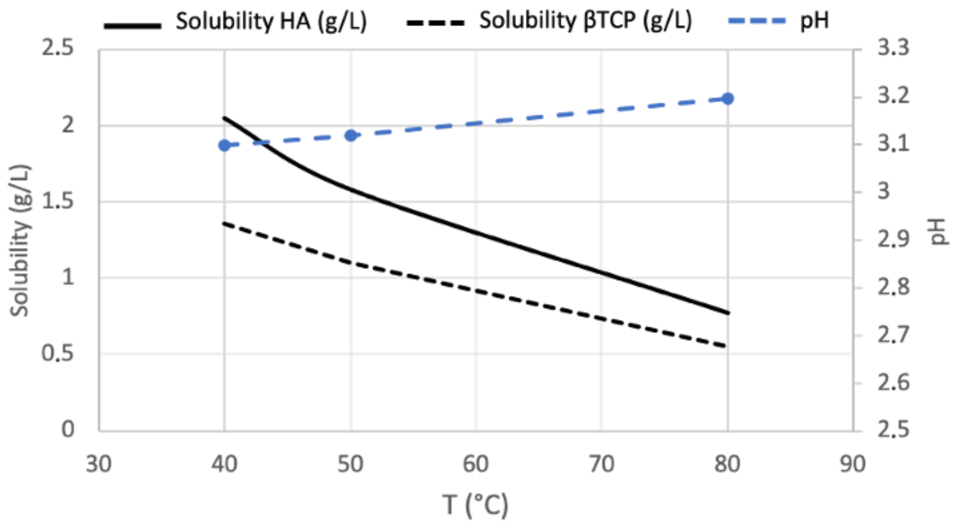

a.

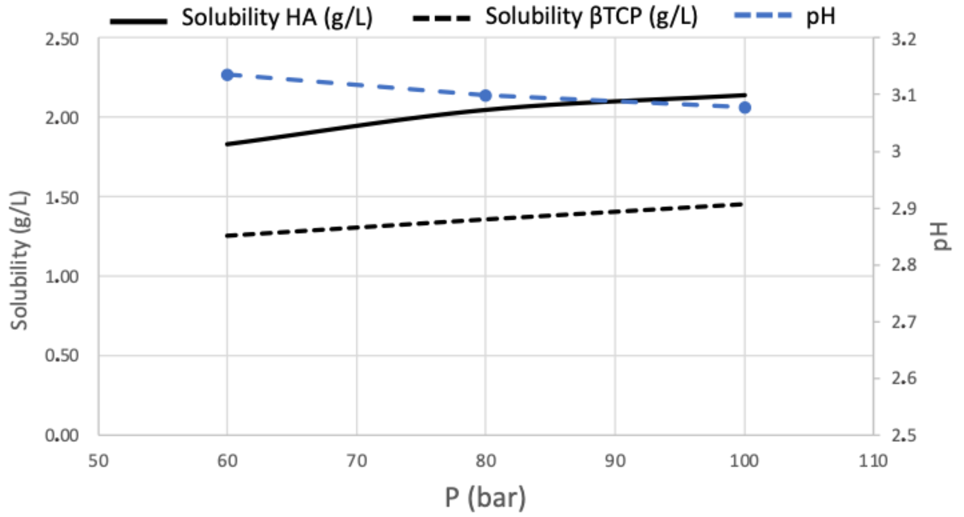

b.

Figure S5: SEM observations on the effect of temperature in the CO_2 process at 80 bar

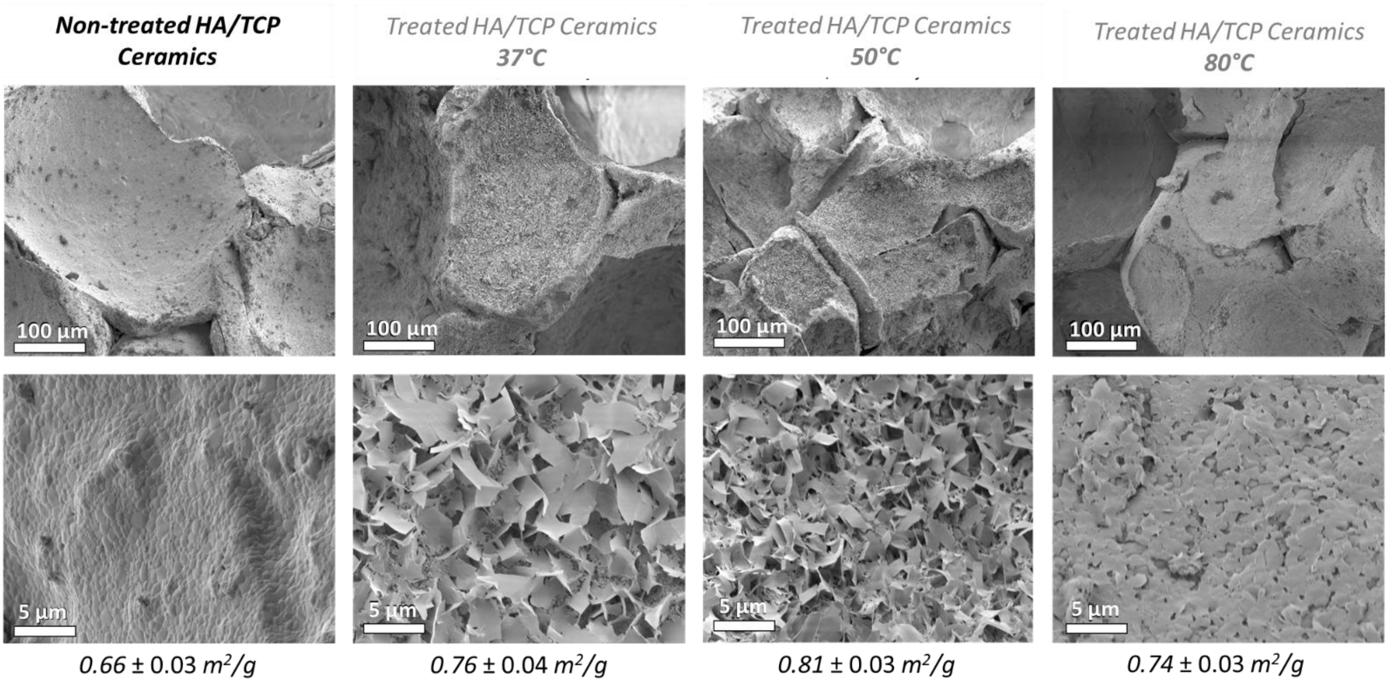

Figure S6: SEM observations on the effect of pressure in the CO_2 process

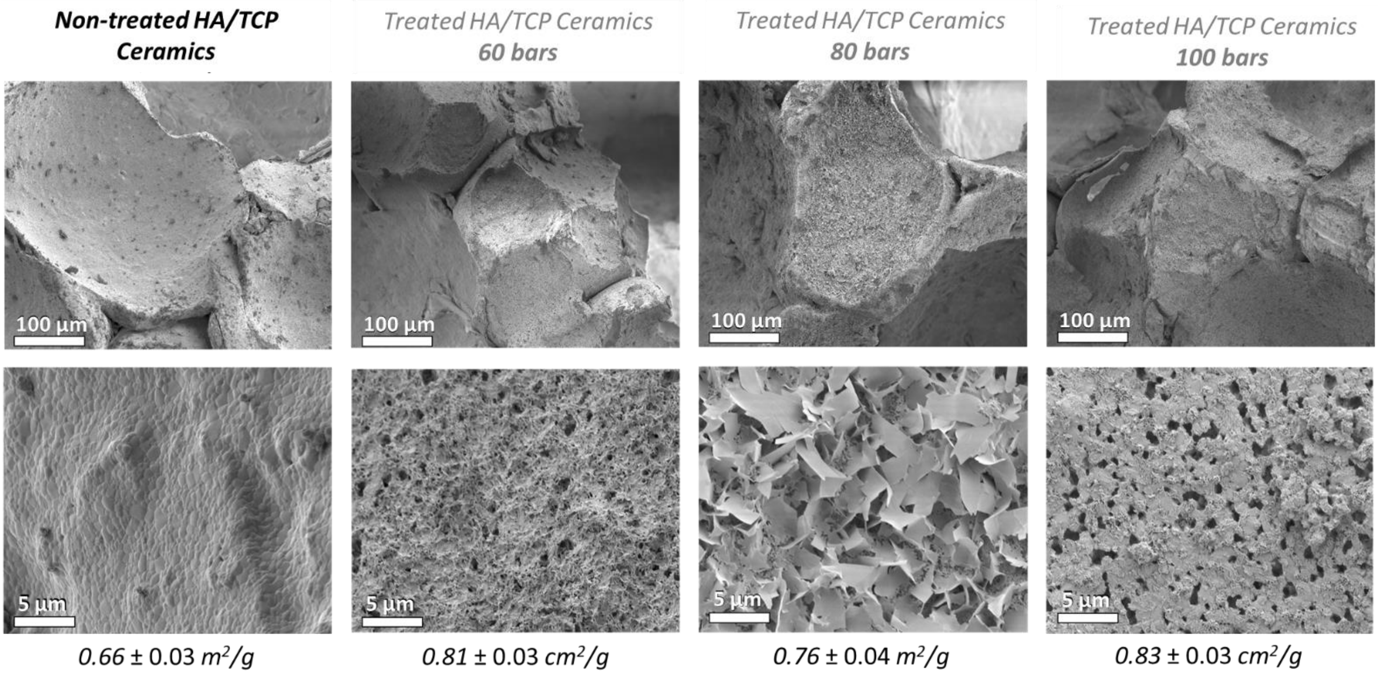

Figure S7: SEM observations on the effect of time under pressure in the CO_2 process

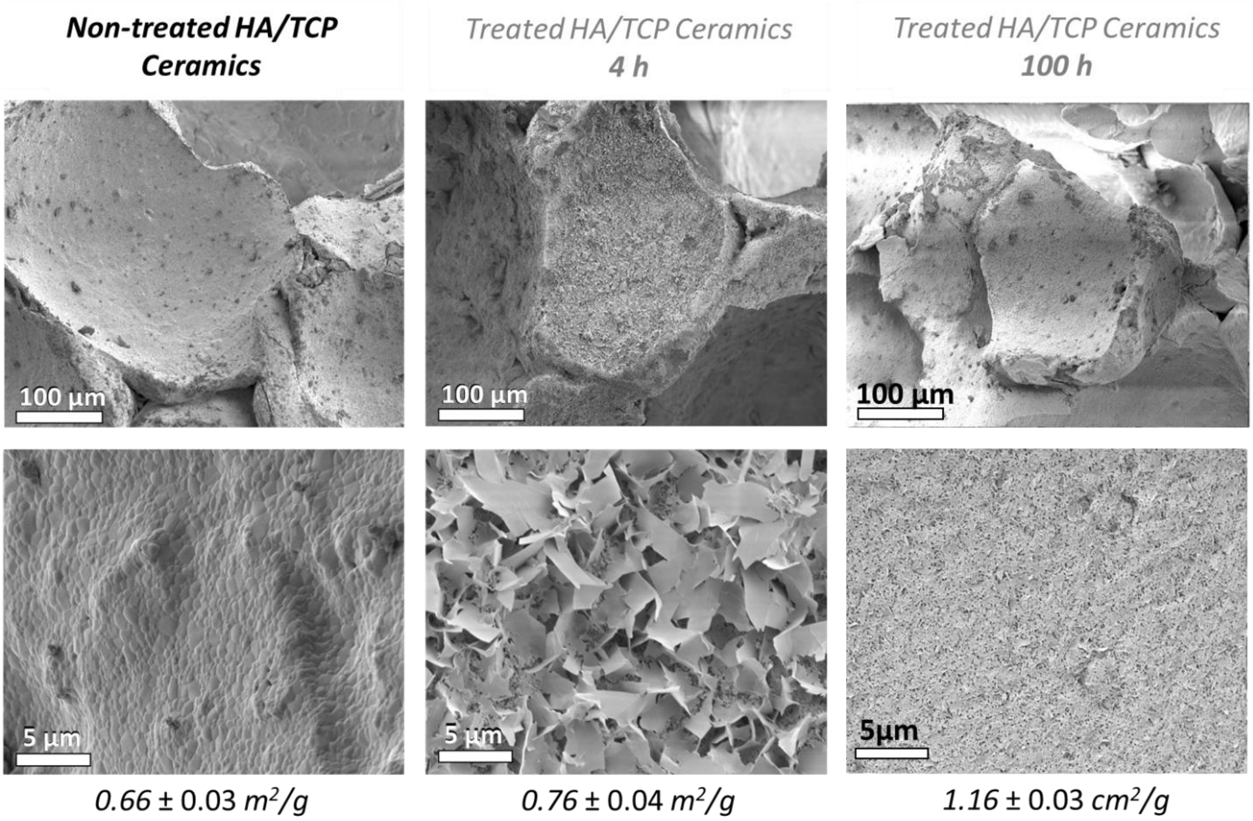

Figure S8: SEM observations on the effect of the L/S ratio in the CO_2 process

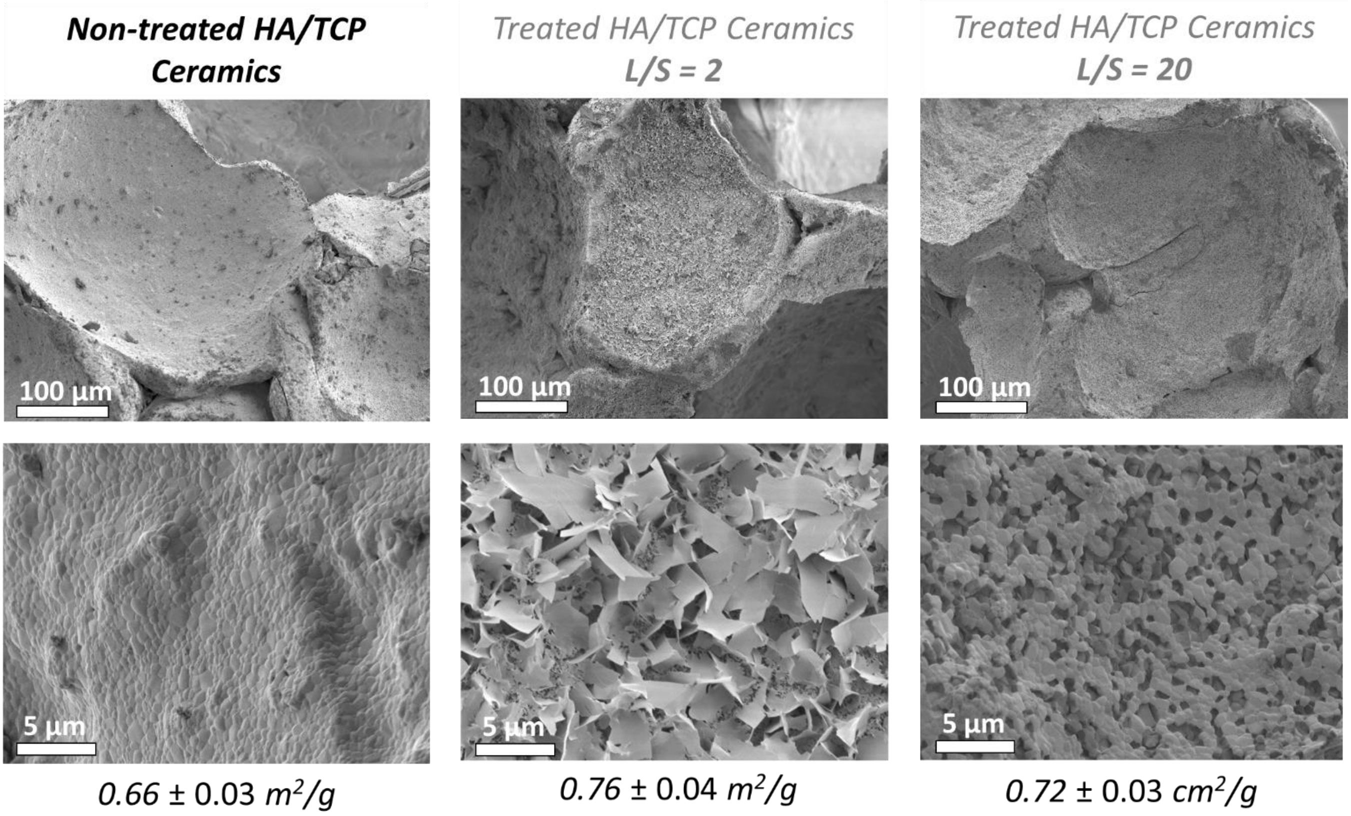

Figure S9: SEM observations on the effect of the depressurization (outgassing) time in the CO_2 process

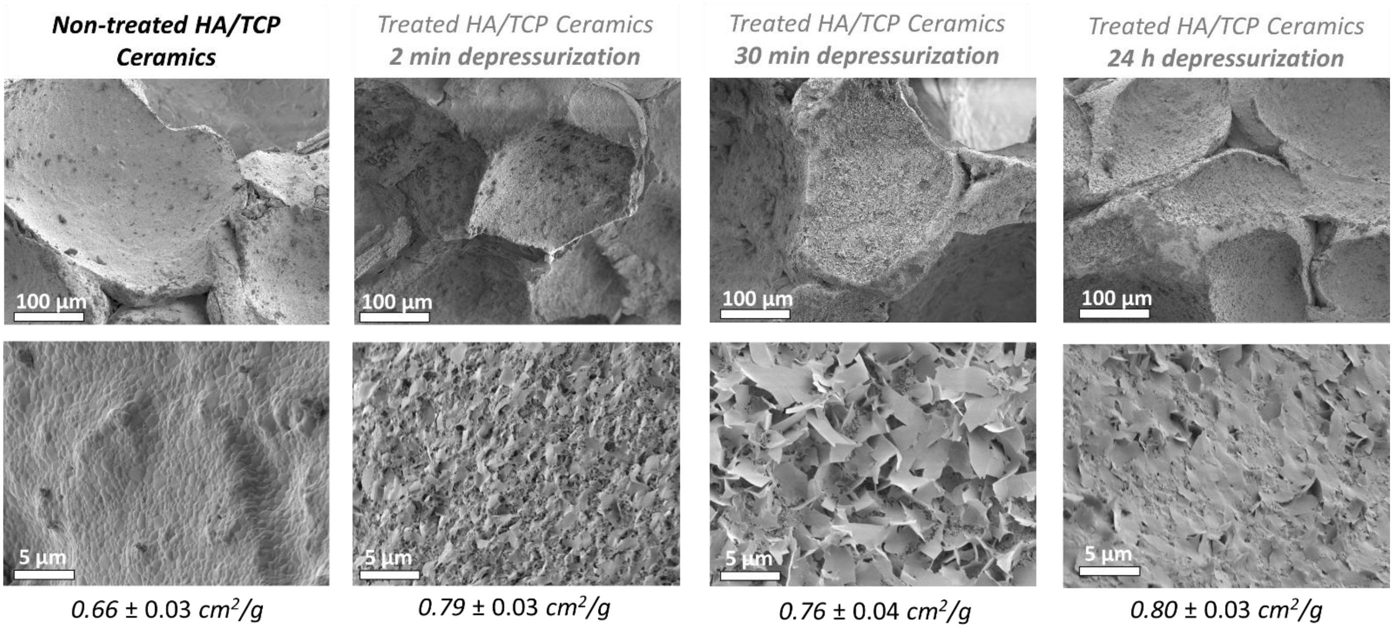

Figure S10: PHREEQC calculations: effect of Cu^{2+} content on the pH at CO_2 saturation at 37°C and 80 bar pressure.

Acetate concentration(g/L)	pH of the CO_2 satured solution
Deionized water	3.098
Cu Min	3.612
Cu Int	3.842
Cu Max	4.061

Figure S11: SEM observation of CO_2-treated scaffold with different concentrations of copper

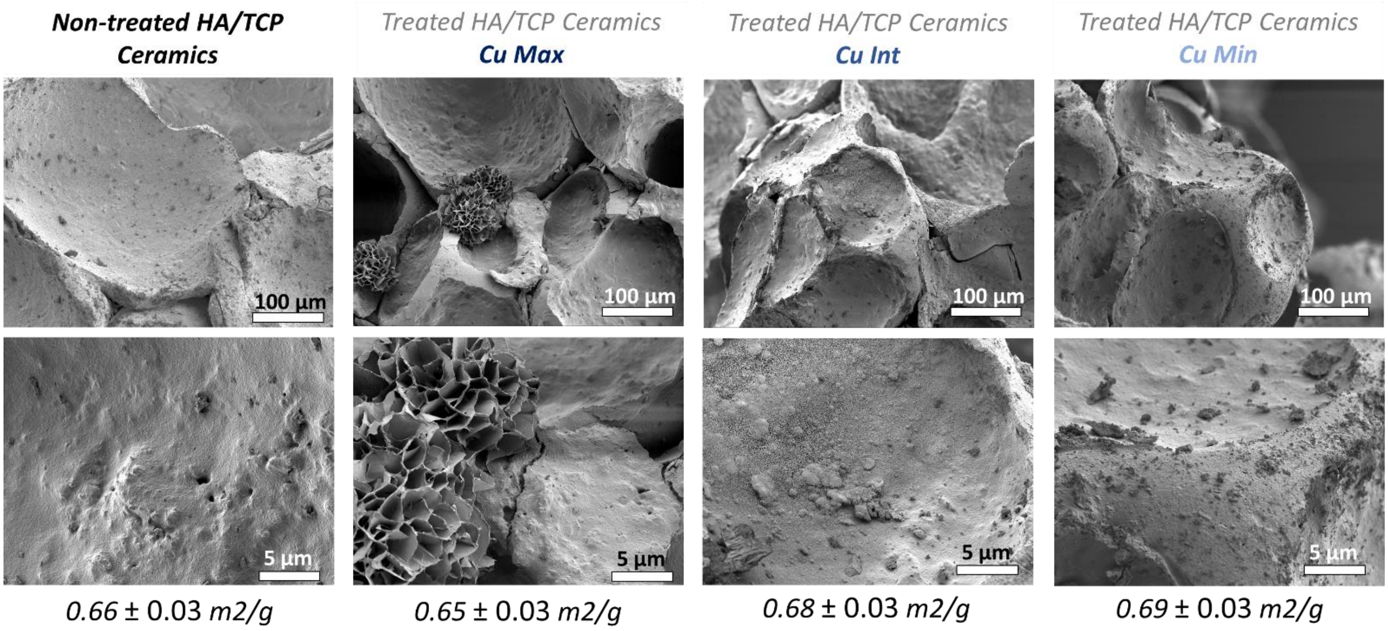

Figure S12: EDX analyses on “Cu Max” modified scaffolds

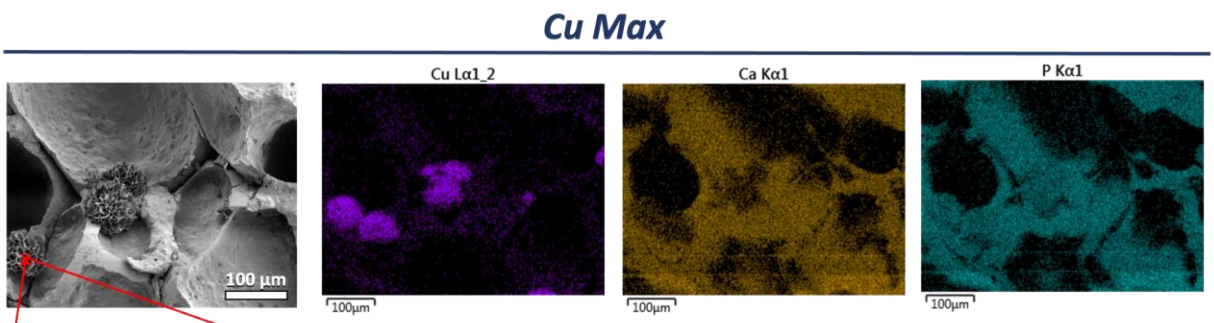

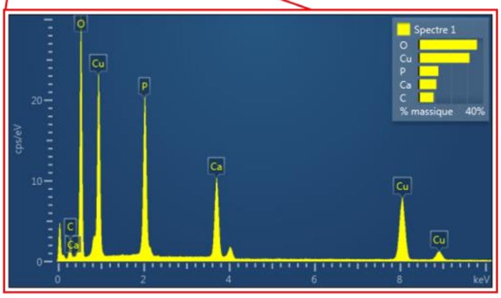

	Average % massique	Standard deviation
C	8.6(7)	0.5(8)
O	38.5(0)	2.1(8)
Ca	12.3(3)	1.5(3)
Cu	30.3(3)	2.0(8)
P	12.0(0)	1.0(0)
